# P-168. Ten Year Evaluation of the Epidemiology of Oral Transmission of Acute Chagas Disease in Brazil

**DOI:** 10.1093/ofid/ofaf695.392

**Published:** 2026-01-11

**Authors:** Alana Pinheiro Alves, Kyle G Crooker, Marcus Zervos, Julius C Mendes Soares Monteiro, Gina Maki

**Affiliations:** Henry Ford Health, Farmington Hills, MI; Henry Ford Hospital, Detroit, MI; Henry Ford Hospital, Detroit, MI; Hospital Universitario Joao de Barros Barreto, Belem, Para, Brazil; Henry Ford Health System, Detroit, MI

## Abstract

**Background:**

Acute Chagas (AC) disease poses a considerable health challenge in Brazil, and many cases are believed to stem from oral transmission (OT). We seek to outline the epidemiology, demographics, and modes of transmission over a ten year period in Brazil, while also examining the pathophysiology of oral transmission.

Brazil's five geopolitical regions

**Figure 1.**
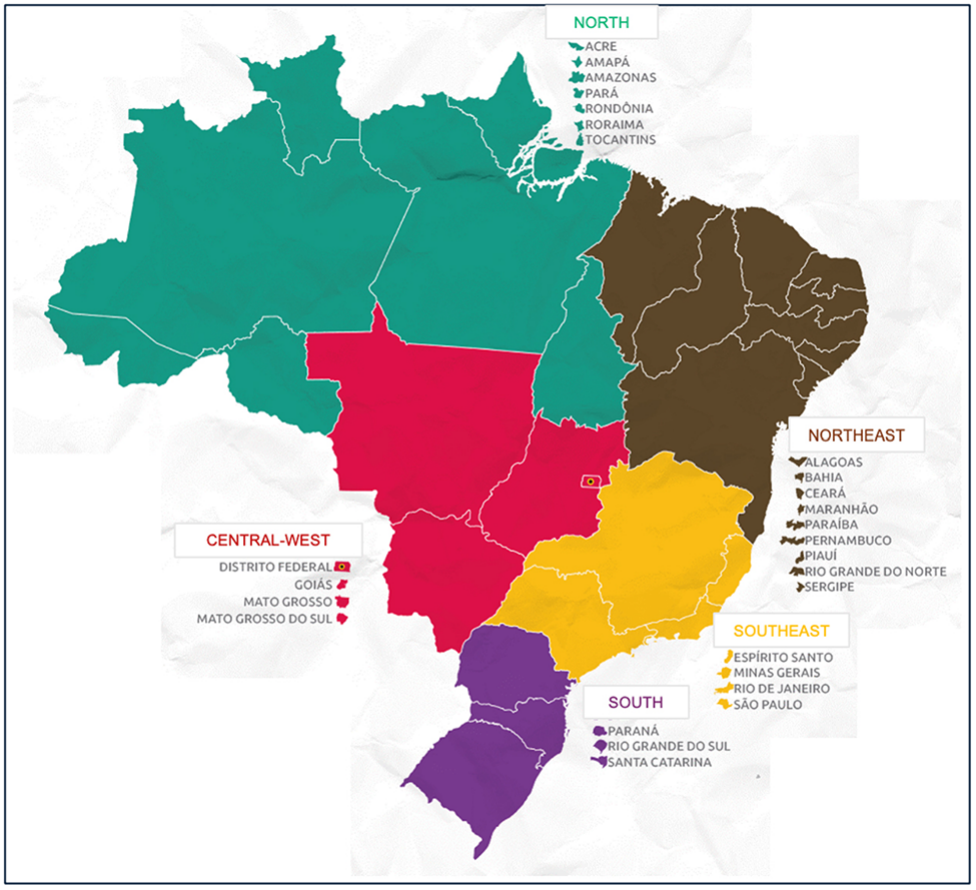
Geopolitical regions of Brazil as of 2014, along with their respective states. From: IBGE (Brazilian Institute of Geography and Statistics), 2019

**Figure:**
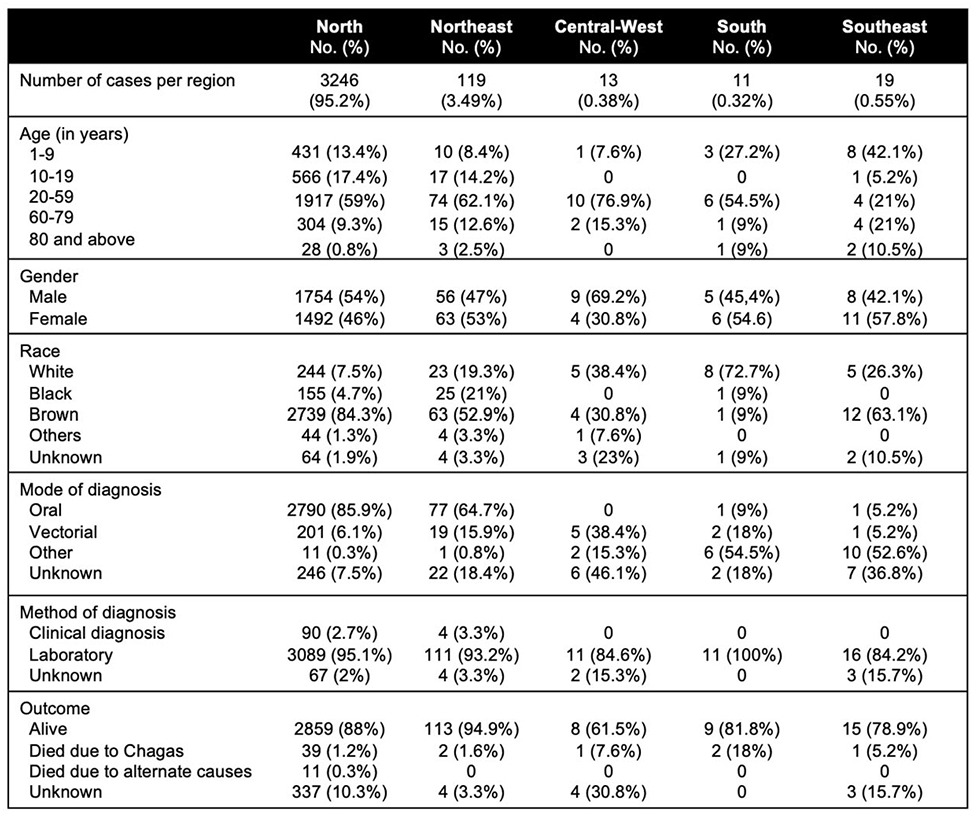

**Methods:**

This retrospective study used data from SINAN, Brazil’s Notifiable Disease Information System, which collects information from the national healthcare system. We analyzed demographic and epidemiological data from Brazil’s five regions between 2014 and 2023 (Figure 1).

**Figure 2.**
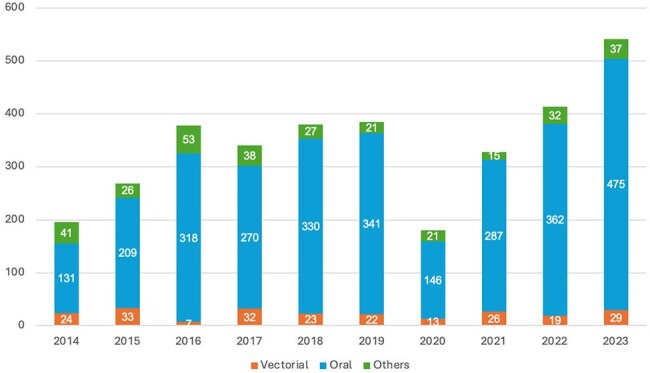
Presumed mode of transmission of Acute Chagas Disease in Brazil, per year, from 2014-2023

**Figure 3.**
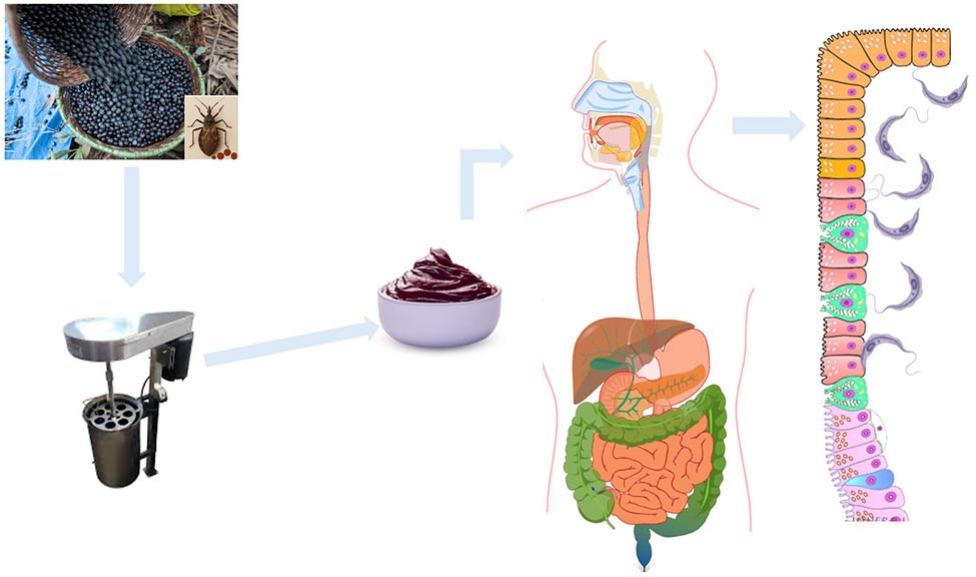
Oral transmission of Chagas disease via ingestion of contaminated açaí

As illustrated in the upper left portion of the figure, triatomine bugs infest açaí fruits after they are harvested. These fruits are typically transported to a processing center, where they are blended into a thick paste or juice for human consumption. When the triatomine is crushed during this process, the trypomastigotes from its gastrointestinal tract are released. Humans then consume the juice, allowing the T. cruzi parasite to penetrate the bloodstream through the gastric mucosa.

**Results:**

We identified a total of 3,408 cases of AC (table 1). Of these, 3,246 were from the Northern region, with most cases (84.1%) occurring in the state of Para. The Southern region reported the fewest cases. The majority of patients were 10 to 19 years of age (1917 cases, 56%). However, nearly half of the cases in the Southeast region involved children under the age of 9 (42%). The primary mode of transmission was presumed to be oral in 2,869 cases (84.1%) (table 2). Notably, for 7 of 19 patients in the Southeast region, the transmission was reported as vertical. Laboratory diagnosis was confirmed for 3,238 patients (95%). Unfortunately, 45 patients died due to complications from AC, with most of these fatalities occurring in the Northern region.

**Conclusion:**

This review highlights the frequency of oral route of transmission of Chagas disease in Brazil and its associated implications, with large impact on the communities within the Amazon rainforest. The pathophysiology of OT occurs when people consume food or beverages contaminated with the feces of triatomines or secretions from parasite reservoirs. In Northern Brazil, a common beverage associated with outbreaks is 'açai' (see figure 3). We suspect this food source has large contribution to OT cases. It is difficult however to confirm oral route, as triatomines can infest areas where food and drinks are contaminated, leading to potential exposure through both oral ingestion and vector-borne transmission. With this review, we hope to highlight the importance of considering oral exposure as a risk factor for AC.

**Disclosures:**

Marcus Zervos, MD, merck: Honoraria

